# Individual heat map assessments demonstrate vestronidase alfa treatment response in a highly heterogeneous mucopolysaccharidosis VII study population

**DOI:** 10.1002/jmd2.12043

**Published:** 2019-06-26

**Authors:** Christine Haller, Wenjie Song, Tricia Cimms, Chao‐Yin Chen, Chester B. Whitley, Raymond Y. Wang, Mislen Bauer, Paul Harmatz

**Affiliations:** ^1^ Department of Clinical Development (Haller), Department of Biometrics (Song), Department of Clinical Outcomes and Research Evaluations (Cimms) Ultragenyx Pharmaceutical Inc. Novato California; ^2^ Department of Pediatrics, and Experimental and Clinical Pharmacology University of Minnesota Minneapolis Minnesota; ^3^ Multidisciplinary Lysosomal Storage Disorder Program Children's Hospital of Orange County Orange California; ^4^ Clinical Genetics Miami Children's Hospital Miami Florida; ^5^ Pediatric Clinical Research Center UCSF Benioff Children's Hospital Oakland Oakland California

**Keywords:** enzyme replacement therapy, heat map, MPS VII, mucopolysaccharidosis, Sly syndrome, vestronidase alfa

## Abstract

Mucopolysaccharidosis (MPS) VII is an ultra‐rare, progressively debilitating, life‐threatening lysosomal disease caused by deficiency of the enzyme, β‐glucuronidase. Vestronidase alfa is an approved enzyme replacement therapy for MPS VII. UX003‐CL301 was a phase 3, randomized, placebo‐controlled, blind‐start study examining the efficacy and safety of vestronidase alfa 4 mg/kg intravenously administered every 2 weeks to 12 patients with MPS VII. Due to the rarity of disease, broad eligibility criteria resulted in a highly heterogeneous population with variable symptoms. For an integrated view of the diverse data, the changes from baseline (or randomization for the placebo period) in clinical endpoints were grouped into three functional domains (mobility, fatigue, and fine motor + self‐care) and analyzed post‐hoc as subject‐level heat maps. Mobility assessments included the 6‐minute walk test, 3‐minute stair climb test, Bruininks‐Oseretsky test (BOT‐2) gross motor function subtests, and patient‐reported outcome assessments (PROs) related to movement, pain, and ambulation. Fatigue assessments included the Pediatric Quality of Life Multidimensional Fatigue Scale and other fatigue‐related PROs. Fine motor + self‐care assessments included BOT‐2 fine motor function subtests and PROs for eating, dressing, hygiene, and caregiver assistance. Most subjects showed improvement in at least one domain. Two subjects improved in two or more domains and two subjects did not show clear improvement in any domain. Both severely and mildly affected subjects improved with vestronidase alfa in clinical assessments, PRO results, or both. Heat map analysis demonstrates how subjects responded to treatment across multiple domains, providing a useful visual tool for studying rare diseases with variable symptoms.

## 1 INTRODUCTION

Mucopolysaccharidosis (MPS) VII (also known as Sly syndrome) is an ultra‐rare, autosomal recessive, debilitating, and life‐threatening lysosomal disease caused by a deficiency of the β‐glucuronidase (GUSB) enzyme that aids in the degradation of glycosoaminoglycans (GAGs).^1^, ^2^ As a result, GAGs such as dermatan, chondroitin, and heparan sulfate, accumulate in a wide variety of tissues and cause organ dysfunction. MPS VII may present in utero or at birth with hydrops fetalis. Though disease manifestations can vary widely, key symptoms include hepatosplenomegaly, cardiac and pulmonary compromise, severe joint and bone abnormalities, cognitive impairment, corneal clouding, hearing loss, and short stature. Based on a European data set, the estimated worldwide prevalence is 0.01 in 100 000 live births.^3^ Most patients with MPS VII die before the second or third decade of life due to progressive organ dysfunction, with a median age of survival for patients postnatally diagnosed of 42 months.^4^

Enzyme replacement therapy (ERT) has demonstrated efficacy for other MPS disorders.^5^, ^6^, ^7^, ^8^, ^9^ In November 2017, the ERT vestronidase alfa (recombinant human GUSB; rhGUS; UX003) was approved by the FDA to treat pediatric and adult patients with MPS VII^10^; this approval was based on efficacy and safety data from the vestronidase alfa clinical trial program.^11^, ^12^, ^13^ UX003‐CL301 was a phase 3, randomized, placebo‐controlled, blind‐start study in 12 subjects. In this novel design, all subjects received at least 24 weeks of treatment with vestronidase alfa, but began active treatment at one of four pre‐defined time points with pre‐treatment placebo data serving as individual controls.^12^ The primary analysis of this study was published Harmatz et al, demonstrating that vestronidase alfa 4 mg/kg administered intravenously every other week reduced urinary GAG levels by 65% after 24 weeks of treatment and resulted in numerical improvement in a clinical multi‐domain‐responder index (mean + 0.5, *P* = .0527).

Given the extreme rarity of MPS VII, the phase 3 eligibility criteria were broad and resulted in a highly heterogeneous study population with variable clinical manifestations and physical and/or cognitive limitations. Here, we employed a heat map analysis in order to understand the individual impact of treatment in this diverse population. This analysis was proposed to address regulatory authority requests for a subject‐by‐subject analysis for the small and heterogeneous UX003‐CL301 study. Clinical endpoints for each subject were grouped into three functional domains—mobility, fatigue, and fine motor + self‐care—and analyzed as subject‐level heat maps. These domains were selected because they allowed for similar clinical efficacy results to be assessed in aggregate for each subject according to major areas of known disease impairment such as, walking/hip pain, lack of energy, and limited dexterity or independence in daily care. By including both physical assessments and patient‐reported outcome assessment scores, an integrated heat map analysis allowed us to assess the totality of response in each domain.

## 2 METHODS

### 2.1 Study participants

Eligible subjects were 5‐35 years of age and had a confirmed diagnosis of MPS VII based on leukocyte or fibroblast glucuronidase enzyme assay or genetic testing; there was no prerequisite leukocyte or fibrolast glucuronidase enzyme level, as this value was determined by independent labs using different assays and reference ranges. Subjects must have had elevated urinary GAG (≥3× the mean normal age range) at screening and have apparent clinical signs of a lysosomal disease as determined by the investigator. Total urine GAG level was determined by ARUP Laboratories (Salt Lake City, Utah) and the normal age range details are provided in Supporting Information Table S1.

### 2.2 Study design

UX003‐CL301 (NCT02230566; 2014‐005638‐71) was a phase 3, randomized, placebo‐controlled, blind‐start (or single‐crossover), 48‐week study examining the efficacy and safety of vestronidase alfa 4 mg/kg IV in subjects with MPS VII. Subjects were randomized (1:1:1:1) to 1 of 4 groups; each group crossed over from treatment with placebo to treatment with vestronidase alfa at a different pre‐defined time point (after 0, 8, 16, or 24 weeks) to obscure the start of active therapy. The study sponsor, investigators, and subjects were blinded to group assignment. Additional details of the study design from the UX003‐CL301 are reported in Harmatz et al.^12^

### 2.3 Assessments

Assessments included in the heat map analysis of study UX003‐CL301 were categorized into three domains—mobility, fatigue, and fine motor + self‐care (Supporting Information Table S2). All assessments were conducted every 8 weeks. Mobility assessments included: 2‐ and 6‐minute walk test (2MWT, 6MWT)^14^; 3‐minute stair climb test (3MSC)^15^; Bruininks‐Oseretsky test of motor proficiency (BOT‐2) running speed and agility and balance^16^; clinical problem evaluation (CPE) walking and running; MPS health assessment questionnaire (MPS HAQ) walking, stairs, and movement^17^; and PROMIS health assessment questionnaire (PROMIS) pain or childhood health assessment questionnaire (CHAQ) pain depending on the age of the subject.

Fatigue assessments included the CPE fatigue assessment and all sections of the Pediatric Quality of Life (PedsQL) Multidimensional Fatigue Scale.^18^ In PedsQL, sleep/rest fatigue refers to the ability to sleep through the night, wake feeling rested, and the need for naps; general fatigue refers to the impact of fatigue on the ability to perform daily activities; cognitive fatigue refers to the impact of fatigue on attention, memory, and processing speed; and total fatigue is a composite of the Sleep/Rest Fatigue Scale, General Fatigue Scale, and Cognitive Fatigue Scale. Fine motor + self‐care assessments included the BOT‐2 fine motor precision and manual dexterity assessments, CPE fine motor and self‐care assessment, as well as the MPS HAQ dressing, eating and drinking, hygiene, and caregiver assistance assessments.

Assessments included in the primary analysis of UX003‐CL301 are reported in Harmatz et al.^12^ Endpoints for UX003‐CL301 were selected based on qualitative research (patient interviews; unpublished) and previous clinical trials evaluating other forms of MPS.^5^, ^6^, ^7^, ^8^, ^9^ While cognitive impairment was noted in medical history, neurological assessments were not performed during this study, as changes in such assessments likely require a longer treatment duration.

### 2.4 Analysis

The heat map analysis was proposed to address regulatory authority requests for a subject‐by‐subject analysis for the small and heterogeneous UX003‐CL301 study. In this post‐hoc analysis, the change from randomization for the placebo period and the change from baseline (last assessment prior to switching to treatment) for the treatment period was calculated for each study assessment, grouped into the mobility, fatigue, or fine motor + self‐care domain, and analyzed as a subject‐level heat map. Heat map color was determined according to the range of changes observed for all subjects by assessment (Tables 1, 2, 3). The maximum absolute change for each assessment was sub‐divided into seven ranges ‐ one with no change or missing data, three levels of improvement, and three levels of worsening. The maximum level of improvement or worsening for each range interval was defined as ±33%, ±67%, or ± 100% of the maximum absolute change. For example, the maximum absolute change for the 6MWT observed in any subject by treatment week 24 was 105 m; therefore subjects in the lightest blue color improved within +33% of the maximum absolute change (1‐35 m), subjects in the intermediate blue color improved between 34% and 67% (36‐70 m), and subjects in the deepest blue color improved between 68% and 100% (71‐105 m). The heat map analysis is one approach to evaluate treatment response with many endpoints in a population with a wide variety of clinical disease manifestations in a descriptive, graphical presentation, without using statistical models to evaluate treatment effect.

**Table 1:** Heat map scoring range for mobility assessments

Color	6MWT (m): *Δ* = 35 m	2MWT (m): *Δ* = 22 m	CPE‐walking^a^: *Δ* = 2	MPS HAQ‐walking^a^: *Δ* = 2	3MSC (steps): *Δ* = 20	CPE‐stairs^a^: *Δ* = 1	MPS HAQ‐stairs^a^: *Δ* = 3	BOT2‐running: *Δ* = 5	CPE‐running^a^: *Δ* = 1	BOT2‐balance: *Δ* = 5	CPE‐gross^a^: *Δ* = 1	MPS HAQ‐movement^a^: *Δ* = 2	CHAQ‐pain^a^: *Δ* = 17	PROMIS‐pain^a^: *Δ* = 27
	[−105, −71]	[−66, −45]	[−5, −4]	[−5, −4]	[−60, −41]	−3	[−8, −6]	[−13, −9]	−3	[−13, −9]	−3	[−4, −3]	[−49, −33]	[−80, −54]
	[−70, −36]	[−44, −23]	[−3, −2]	[−3, −2]	[−40, −21]	−2	[−5, −3]	[−8, −5]	−2	[−8, −5]	−2	−2	[−32, −17]	[−53, −27]
	[−35, −1]	[−22, −1]	−1	−1	[−20, −1]	−1	[−2, −1]	[−4, −1]	−1	[−4, −1]	−1	−1	[−16, −1]	[−26, −1]
	0	0	0	0	0	0	0	0	0	0	0	0	0	0
	[1, 35]	[1, 22]	1	1	[1, 20]	1	[1, 2]	[1, 4]	1	[1, 4]	1	1	[1, 16]	[1, 26]
	[36, 70]	[23, 44]	[2, 3]	[2, 3]	[21, 40]	2	[3, 5]	[5, 8]	2	[5, 8]	2	2	[17, 32]	[27, 53]
	[71, 105]	[45, 66]	[4, 5]	[4, 5]	[41, 60]	3	[6, 8]	[9, 13]	3	[9, 13]	3	[3, 4]	[33, 49]	[54, 80]

*Note*: White cells (not shown in table, but included in Figure 1) indicate that the assessment was not completed.

Abbreviations: 2MWT, 2‐minute walk test; 3MSC (steps), 3‐minute stair climb test; 6MWT, 6‐minute walk test; BOT2, Bruininks‐Oseretsky test of motor proficiency; CHAQ, childhood health assessment questionnaire; CPE, clinical problem evaluation; MPS HAQ, MPS health assessment questionnaire; PROMIS, PROMIS health assessment questionnaire.

a The negative changes (from randomization for placebo period and from baseline for UX003 period) are displayed since decreases in these scores indicate improvement.

**Table 2:** Heat map scoring range for fatigue assessments

Color	Total fatigue: *Δ* = 11	General fatigue: *Δ* = 14	Sleep/rest fatigue: *Δ* = 13	Cognitive fatigue: *Δ* = 18	CPE‐fatigue^a^: *Δ* = 2
	[−32, −22]	[−42, −29]	[−38, −26]	[−52, −35]	[−5, −4]
	[−21, −11]	[−28, −15]	[−25, −13]	[−34, −18]	[−3, −2]
	[−10, −1]	[−14, −1]	[−12, −1]	[−17, −1]	−1
	0	0	0	0	0
	[1, 10]	[1, 14]	[1, 12]	[1, 17]	1
	[11, 21]	[15, 28]	[13, 25]	[18, 34]	[2, 3]
	[22, 32]	[29, 42]	[26, 38]	[35, 52]	[4, 5]

*Note*: White cells (not shown in table, but included in Figure 1) indicate that the assessment was not completed.

Abbreviation: CPE, clinical problem evaluation.

a The negative changes (from randomization for placebo period and from baseline for UX003 period) are displayed since decreasing in these scores indicates improvement.

**Table 3:** Heat map scoring range for fine motor and dexterity assessments

Color	BOT2‐fine: *Δ* = 3	BOT2‐dexterity: *Δ* = 2	CPE‐fine motor^a^: *Δ* = 1	CPE‐self care^a^: *Δ* = 1	MPS HAQ‐dressing^a^: *Δ* = 2	MPS HAQ‐eating and drinking^a^: *Δ* = 1	MPS HAQ‐hygiene^a^: *Δ* = 1	MPS HAQ‐caregiver assist^a^: *Δ* = 5
	[−7, −5]	[−6, −5]	−2	−3	[−6, −5]	−3	−3	[−14, −10]
	[−4, −3]	[−4, −3]	−1	−2	[−4, −3]	−2	−2	[−9, −5]
	[−2, −1]	[−2, −1]		−1	[−2, −1]	−1	−1	[−4, −1]
	0	0	0	0	0	0	0	0
	[1, 2]	[1, 2]		1	[1, 2]	1	1	[1, 4]
	[3, 4]	[3, 4]	1	2	[3, 4]	2	2	[5, 9]
	[5, 7]	[5, 6]	2	3	[5, 6]	3	3	[10, 14]

*Note*: White cells (not shown in table, but included in Figure 1) indicate that the assessment was not completed.

Abbreviations: BOT2, Bruininks‐Oseretsky test of motor proficiency; CPE, clinical problem evaluation; MPS HAQ, MPS health assessment questionnaire.

a The negative changes (from randomization for placebo period and from baseline for UX003 period) are displayed since decreasing in these scores indicates improvement.

Due to the rarity of MPS VII and the small number of subjects in this trial, individual patient data will not be shared in order to safeguard patient privacy.

## 3 RESULTS

### 3.1 Baseline

A total of 14 subjects were screened and two subjects withdrew consent before initiating treatment. Twelve subjects (4 males, 8 females) enrolled, completed the study participation at US sites, and were included in the final analysis. Seven subjects had a confirmed diagnosis of MPS VII with genetic screening and the remaining five subjects had a confirmed diagnosis based on a leukocyte or fibroblast glucuronidase enzyme assay. Subjects ranged from 8 to 25 years old (nine subjects were <18 years old) and were from the US, Mexico, Brazil, or Portugal. There were a total of two missed doses in two separate subjects, one dose of vestronidase alfa at week 42 and one dose of placebo at week 6.

Baseline demographics and characteristics demonstrated a heterogeneous study population. Baseline evaluations for assessments included in the heat map analysis are shown in Supporting Information Table S3. All subjects had abnormalities in their medical history including: musculoskeletal and connective tissue disorders (91.7% of subjects); nervous system disorders (83.3%); cardiac disorders (75.0%); congenital, familial, and genetic disorders (75.0%); general disorders and administration site conditions (75.0%); infections and infestations (75.0%); respiratory, thoracic and mediastinal disorders (75.0%); hepatobiliary disorders (66.7%); skin and subcutaneous tissue disorders (66.7%); investigations, including increased blood alkaline phosphatase, body weight below normal, cardiac murmur, increased hepatic enzyme, and normal menstruation (58.3%); surgical and medical procedures (58.3%, full list of procedures is provided in Supporting Information Table S4); and gastrointestinal disorders (50.0%).

Pervasive physical and cognitive impairments prevented the assessment of some measures at baseline: three of the 12 subjects could not walk; nine of the 12 were not able to perform pulmonary function tests (PFT); five of the 12 could not perform the BOT‐2 gross motor tests; and five of the 12 could not perform the visual acuity test.

### 3.2 Heat map domain efficacy

**Figure 1:**
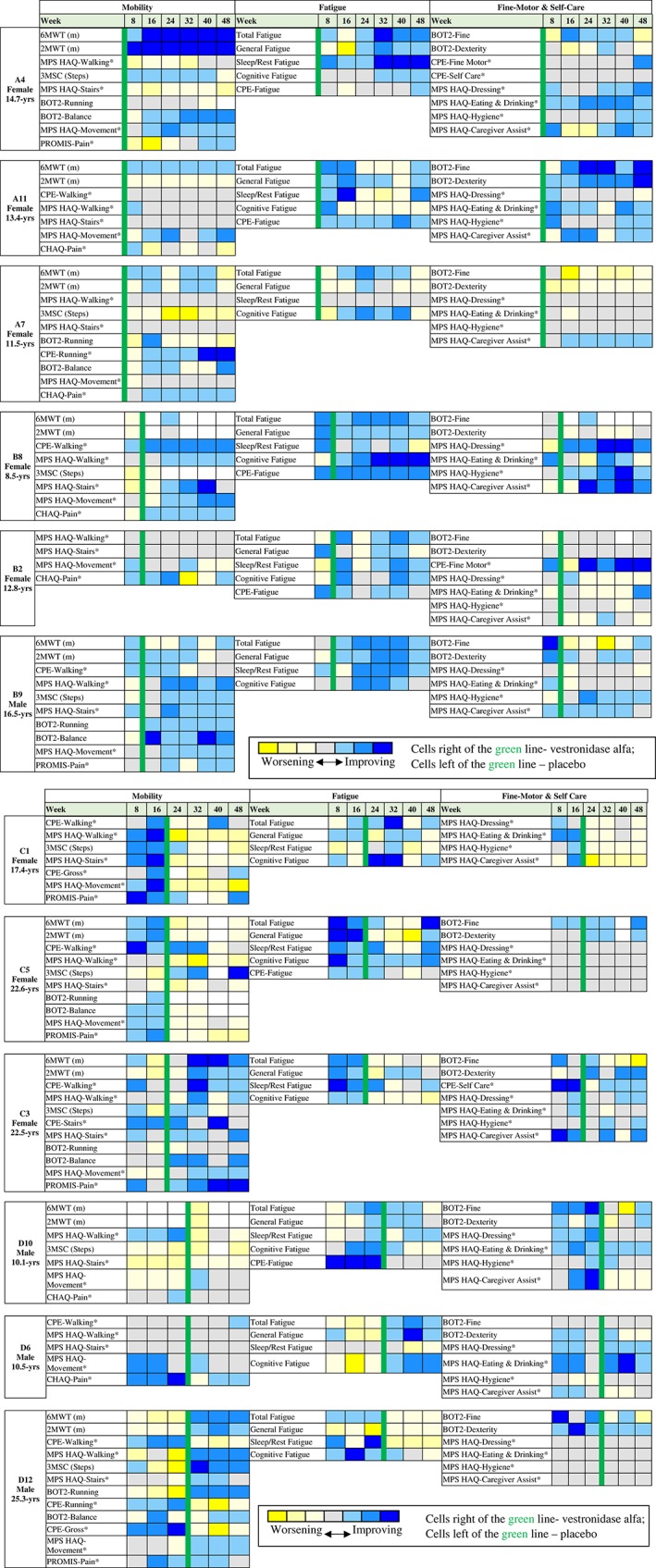
Individual heat map response. The color of each cell is based on the change from randomization during placebo treatment or from baseline during vestronidase alfa treatment; yellow indicates worsening, gray indicates no change, blue indicates improvement, and white indicates when an assessment is unavailable. Each cell represents an 8‐week interval. *The negative changes (from randomization for placebo period and from baseline for UX003 period) are displayed since decreases in these scores indicate improvement. Abbreviations: 2MWT, 2‐minute walk test; 3MSC (steps), 3‐minute stair climb test; 6MWT, 6‐minute walk test; BOT2, Bruininks‐Oseretsky test of motor proficiency; CHAQ, childhood health assessment questionnaire; CPE, clinical problem evaluation; MPS HAQ, MPS health assessment questionnaire; PROMIS, PROMIS health assessment questionnaire

The level of treatment response across domains for each individual subject is demonstrated in Figure 1. Ten of the 12 enrolled subjects showed improvement (predominance of blue shading) after crossing over to receive vestronidase alfa in at least one of the three heat map domains.

#### 3.2.1 Mobility

Subjects A4, C3, and D12 demonstrated clear improvements in more than one assessment in the mobility domain. After receiving 24 weeks of active treatment, subject A4 showed an increase of 83 m in the 6MWT, a reduction in the total number of rest stops during the 6MWT, an increase of 11 steps in the 3MSC test, and improvement in a timed task for stepping sideways over a beam. Both C3 and D12 showed declines in distance walked in the 6MWT during the placebo period, but increases in distance walked after beginning treatment with vestronidase alfa. These two subjects also saw improvements in other mobility domains such as balance or climbing stairs.

Subjects A7, B8, and B9 also showed some improvement in the mobility domain. For example, subject B9 increased the number of stairs climbed in 3 minutes and was able to step sideways over a beam, which was not possible prior to treatment with vestronidase alfa. Nevertheless, this same subject showed little change in the 6MWT. The remaining six subjects showed little to no improvement in the mobility domain. It should be noted that a majority of the six remaining subjects had advanced disease with physical and/or cognitive impairment at baseline, including two subjects that required the use of a wheelchair; these cognitive and physical impairments likely limited their ability to comprehend test instructions and reliably complete the mobility tasks.

#### 3.2.2 Fatigue

Subjects A4, B8, B9, B2, and D6 showed substantial improvements in multiple fatigue assessments. Though subjects B8, B2, and B9 saw some improvement during treatment with placebo, their fatigue scores continued to improve after crossing over to receive vestronidase alfa and were sustained through the active treatment phase. After 24 weeks of treatment, large increases (corresponding to improvement) in fatigue scores, most notably in cognitive fatigue, brought subject B8 into the range reported for that of healthy children.^19^, ^20^

Four subjects, A11, A7, C1, and C5, showed variable improvement in fatigue. Subject A7 reported no difficulty with sleep‐related fatigue at baseline, which did not change with treatment, but showed substantial improvement in cognitive fatigue. Subject C1 also showed noteworthy improvement in cognitive fatigue despite slight decreases in other fatigue assessments at week 48. The delayed improvement in fatigue scores for subject C5 coincided with the resolution of an acute viral illness. The remaining three subjects showed little to no improvement in the fatigue domain; however, two of these subjects showed clear improvement in the mobility domain.

#### 3.2.3 Fine motor + self‐care

Subjects A11 and B8 showed clear improvement in multiple assessments within the fine motor + self‐care domain, with both subjects showing substantial improvement in the assessments that often required caregiver assistance. Subject B8 was unable to complete the BOT‐2 assessments due to cognitive impairment, but all 12 of the 18 MPS HAQ items that scored “extreme difficulty” improved after 24 weeks of treatment with vestronidase alfa.

Four subjects, A4, C5, C3, and B9, showed some improvement in fine motor + self‐care assessments. Subject A4 demonstrated small improvements in both fine motor and self‐care assessments, more notably after 24 weeks of treatment with vestronidase alfa. Subject C3 also showed small improvements in more than one assessment, but improvement was noted with both placebo and active treatment. Subject C5 saw greater improvement in fine motor and dexterity assessments, while subject B9 displayed greater improvements in the self‐care assessments. The remaining six subjects exhibited little to no change in the fine motor + self‐care domain. Two of these six subjects had baseline scores that indicated minimal impairment in self‐care assessments, therefore there was less potential for improvement.

### 3.3 Safety

A summary of safety findings from this study can be found in Harmatz et al.^12^ Briefly, all subjects experienced at least one treatment‐emergent adverse event (TEAE) during both the placebo and vestronidase alfa treatment periods. There were no deaths or discontinuations from the study. C5 experienced a viral illness at treatment week 24, which may have contributed to her lack of clear improvement in one of the heat map domains.

Two subjects experienced a serious adverse event during the vestronidase alfa treatment period. B8 experienced a treatment‐related averse event of anaphylactoid reaction secondary to an accidentally high bolus infusion rate during the first hour of vestronidase alfa administration; the infusion was stopped and restarted the same day after the event was resolved with treatment of oxygen, epinephrine, hydrocortisone and diphenhydramine. D10 experienced a craniocerebral injury from falling off the bed; this event was considered unrelated to treatment, resolved, and no action was taken with vestronidase alfa treatment.

Overall, there were no significant differences in exposure‐adjusted incidence rates of TEAE, defined as the total number of occurrences of the TEAE divided by the total amount of time subjects participated in the analysis group phase.

## 4 DISCUSSION

MPS VII disease manifestations were highly variable and clinical response to vestronidase alfa showed individual differences across subjects within this study. Overall, the integrated heat map analysis demonstrated that 10 of 12 subjects showed improvement in at least one clinical efficacy domain relevant to their specific disease manifestations. For the mobility domain, half of all subjects displayed improvement in either some or many assessments. In the fatigue domain, five subjects showed clear improvement across assessments and an additional four subjects showed variable improvement. Two subjects demonstrated consistent improvements in the fine motor + self‐care domain, while an additional four subjects showed variable improvement. Lastly, some subjects demonstrated clear improvement in more than one heat map domain, and two subjects did not show clear improvement in any domain.

In some cases where there was little to no improvement, advanced disease at baseline, such as developmental delay or physical impairment, prevented some subjects from following test instructions or completing tasks. In contrast, some subjects displayed little impairment at baseline in a particular task, such as the self‐care assessment of eating, and therefore there was little room for improvement. The heat map analysis provided a clear integrated appreciation of how subjects improved in symptoms relevant to how they experience MPS VII—a disease with a highly variable set of manifestations.

The wide range of clinical manifestations of disease observed in patients in this study was consistent with previous reports of patients with MPS VII.^1^ It will be important to follow these patients to evaluate long‐term treatment, and compare clinical outcomes with natural history data from patients with MPS VII. Data from the ongoing long‐term extension study (UX003‐CL202; NCT02432144) and the Disease Monitoring Program (UX003‐CL401; NCT03604835) in patients with MPS VII with and without vestronidase alfa treatment will help to provide a long‐term understanding of disease progression and response to treatment.

This analysis represents one approach to evaluating treatment response in a study with many endpoints, and did not use statistical significance to evaluate treatment effect. The grouping of these assessments was based on three specific domains. Of note, these assessments could be determined prospectively and used in different, related domains; or new assessments could be used to evaluate other domains. This analysis may be limited by the narrow margin that defined no change as an absolute change of zero, compared to the varying levels of improvement and worsening defined as within ±33%, ±67%, or ±100% of the maximum absolute change. Because the range was determined by the maximum absolute change, there is no clinical definition for the equivalent range of each assessment. Nevertheless, this approach is meant to be visually interpreted at a high level, across multiple related endpoints, on a per‐subject basis. The ranges can also be adapted to integrate even more levels of improvement or worsening. The seven levels, including no change, used in this heat map analysis were sufficient to detect a varied response to treatment across subjects. For the assessment of long‐term therapeutic use, the heat map analysis can be used to assess change from baseline over the course of several months. For a more complete long‐term analysis, it is likely most effective to use the heat map analysis in conjunction with other tools, such as comparing natural history data to that of patients treated with vestronidase alfa.

Importantly, the heat map analysis was not intended to determine the overall impact for all subjects in the study, but rather provide an understanding of how treatment with vestronidase alfa affected each individual's experience of MPS VII. Such an approach is valuable in conjunction with additional analyses, when assessing heterogeneous diseases that may present and progress differently in each individual, like MPS VII. A subgroup analysis was not considered appropriate for this study because of the small number of subjects included (N = 12). Any subgroup analysis in this small heterogeneous population would be confounded by including some patients in an assessment for which they have little to no impairment at baseline with some patients that have great baseline impairment.

This integrated heat map analysis demonstrates how subjects responded to treatment across multiple domains, providing a useful visual tool for studying complex genetic diseases that present with multiple, variable symptoms. While not all subjects improved in one specific domain, this visualization approach enabled assessment of how treatment improved clinical outcomes that were inter‐related and relevant to each individual's disease manifestations. This tool could be applied in other rare disease clinical trials including case studies to evaluate the totality of clinical baseline and outcome data on an individual subject basis.

## AUTHOR CONTRIBUTIONS

C.H., W.S., T.C., and C.‐Y.C.: study design, data acquisition, data analyses, data interpretation, and drafting of the manuscript. C.B.W., R.Y.W., M.B., and P.H.: data acquisition, data interpretation, and drafting of the manuscript.

## COMPETING INTEREST

C.H., W.S., T.C., and C.‐Y.C. are employees and shareholders of Ultragenyx Pharmaceutical Inc. C.B.W., R.Y.W., M.B., and P.H. have served as clinical investigators in clinical trials with the product manufactured by Ultragenyx Pharmaceutical Inc. The content of the article has not been influenced by the sponsors. Dr. Harmatz has received support for this project from the National Center for Advancing Translational Sciences, National Institutes of Health (NIH), through UCSF‐CTSI grant number UL1 TR000004. The contents of this article are solely the responsibility of the authors and do not necessarily represent the official views of the NIH.

## DETAILS OF ETHICS APPROVAL

Institutional review boards/ethics committees approved the protocol. Parents or guardians provided written informed permission for children to participate, and when appropriate, the subjects' assents were obtained before participating. An external data monitoring committee monitored patients' safety and efficacy during the study. The trial is registered with ClinicalTrials.gov (NCT02230566) and the EU Register (2014‐005638‐71).

## PATIENT CONSENT STATEMENT

Parents or guardians provided written informed permission for children to participate, and when appropriate, the subjects' assents were obtained before participating.

## DOCUMENTATION OF APPROVAL FROM THE INSTITUTIONAL COMMITTEE FOR CARE AND USE OF LABORATORY ANIMALS (OR COMPARABLE COMMITTEE)

Institutional review boards/ethics committees, operating in accordance with Title 21 Code of Federal Regulations Part 56, approved the protocol for study UX023‐CL301 (NCT02230566).

## Supporting information


**Data S1 Table S1.** Total urine glycosaminoglycans concentration (mg/mmol creatinine)
**Table S2.** Domains for heat map analysis
**Table S3.** Individual baseline results for assessments included in heat map analysis
**Table S4.** List of preferred terms included in surgical and medical procedures
**Figure S1.** Individual heat map response with numbers

## References

[1] Montano AM , Lock‐Hock N , Steiner RD , et al. Clinical course of Sly syndrome (mucopolysaccharidosis type VII). J Med Genet. 2016;53:403‐418. 26908836 10.1136/jmedgenet-2015-103322 PMC4893087

[2] Muenzer J . Overview of the mucopolysaccharidoses. Rheumatology. 2011;50(suppl 5):v4‐v12. 22210669 10.1093/rheumatology/ker394

[3] Orphanet Report Series 2019 https://www.orpha.net/orphacom/cahiers/docs/GB/Prevalence_of_rare_diseases_by_alphabetical_list.pdf.

[4] Zielonka M , Garbade SF , Kolker S , Hoffmann GF , Ries M . Quantitative clinical characteristics of 53 patients with MPS VII: a cross‐sectional analysis. Genet Med. 2017;9(9):983‐988. 10.1038/gim.2017.10 28383542

[5] Harmatz P , Giugliani R , Schwartz I , et al. Enzyme replacement therapy for mucopolysaccharidosis VI: a phase 3, randomized, double‐blind, placebo‐controlled, multinational study of recombinant human N‐acetylgalactosamine 4‐sulfatase (recombinant human arylsulfatase B or rhASB) and follow‐on, open‐label extension study. J Pediatr. 2006;148:533‐539. 16647419 10.1016/j.jpeds.2005.12.014

[6] Hendriksz CJ , Burton B , Fleming TR , et al. Efficacy and safety of enzyme replacement therapy with BMN 110 (elosulfase alfa) for Morquio A syndrome (mucopolysaccharidosis IVA): a phase 3 randomised placebo‐controlled study. J Inherit Metab Dis. 2014;37(6):979‐990. 24810369 10.1007/s10545-014-9715-6 PMC4206772

[7] Kakkis ED , Muenzer J , Tiller GE , et al. Enzyme‐replacement therapy in mucopolysaccharidosis I. New Engl J Med. 2001;344:182‐188. 11172140 10.1056/NEJM200101183440304

[8] Muenzer J , Wraith JE , Beck M , et al. A phase II/III clinical study of enzyme replacement therapy with idursulfase in mucopolysaccharidosis II (Hunter syndrome). Genet Med. 2006;8:465‐473. 16912578 10.1097/01.gim.0000232477.37660.fb

[9] Wraith JE , Clarke LA , Beck M , et al. Enzyme replacement therapy for mucopolysaccharidosis I: a randomized, double‐blinded, placebo‐controlled, multinational study of recombinant human alpha‐l‐iduronidase (laronidase). J Pediatr. 2004;144:581‐588. 15126990 10.1016/j.jpeds.2004.01.046

[10] FDA (2017) FDA approves treatment for rare genetic enzyme disorder. https://www.fda.gov/NewsEvents/Newsroom/PressAnnouncements/ucm585308.htm. Accessed May 29, 2019.

[11] Fox JE , Volpe L , Bullaro J , Kakkis ED , Sly WS . First human treatment with investigational rhGUS enzyme replacement therapy in an advanced stage MPS VII patient. Mol Genet Metab. 2015;114:203‐208. 25468648 10.1016/j.ymgme.2014.10.017 PMC4360956

[12] Harmatz P , Whitley CB , Wang RY , et al. A novel blind start study design to investigate vestronidase alfa for mucopolysaccharidosis VII, an ultra‐rare genetic disease. Mol Genet Metab. 2018;123(4):488‐494. 29478819 10.1016/j.ymgme.2018.02.006

[13] Jones SA , Ghosh A , Breen C , Kakkis ED , Sly WS . Enzyme replacement therapy (ERT) for mucopolysaccharidosis VII (MPS VII; Sly syndrome) reduces lysosomal storage in a 36‐week phase 1/2 clinical study. Mol Genet Metab. 2015;114:S59.

[14] Geiger R , Strasak A , Treml B , et al. Six‐minute walk test in children and adolescents. J Pediatr. 2007;150:395‐399. 17382117 10.1016/j.jpeds.2006.12.052

[15] Zech A , Steib S , Sportwiss D , Freiberger E , Pfeifer K . Functional muscle power testing in young, middle‐aged, and community‐dwelling nonfrail and prefrail older adults. Arch Phys Med Rehabil. 2011;92:967‐971. 21514567 10.1016/j.apmr.2010.12.031

[16] Bruininks R , Bruininks B . Bruininks‐Oseretsky Test of Motor Proficiency. 2nd ed. Minneaplois, MN: NCS Pearson; 2005.

[17] Hendriksz CJ , Berger KI , Lampe C , et al. Health‐related quality of life in mucopolysaccharidosis: looking beyond biomedical issues. Orphanet J Rare Dis. 2016;11:119‐134. 27561270 10.1186/s13023-016-0503-2 PMC5000418

[18] Varni JW , Seid M , Rode CA . The PedsQL: measurement model for the pediatric quality of life inventory. Med Care. 1999;37:126‐139. 10024117 10.1097/00005650-199902000-00003

[19] Varni JW , Burwinkle TM , Szer IS . The PedsQL Multidimensional Fatigue Scale in pediatric rheumatology: reliability and validity. J Rheumatol. 2004;31:2494‐2500. 15570657

[20] Varni JW , Limbers CA , Bryant WP , Wilson DP . Assessment of fatigue in pediatric patients with short stature utilizing the PedsQL Multidimensional Fatigue Scale. Child Health Care. 2012;41:162‐181.

