# Supplementary material for: Individual heat map assessments demonstrate vestronidase alfa treatment response in a highly heterogeneous mucopolysaccharidosis VII study population
Source: JIMD Rep. 2019 Jun 26;49(1):53–62. doi: 10.1002/jmd2.12043 (PMC6718107; doi:10.1002/jmd2.12043)
Supplement: Supplementary file 1 — Data S1 Table S1. Total urine glycosaminoglycans concentration (mg/mmol creatinine) Table S2. Domains for heat map analysis Table S3. Individual baseline results for assessments included in heat map analysis Table S4. List of preferred terms included in surgical and medical procedures Figure S1. Individual heat map response with numbers [file JMD2-49-53-s001.docx]

Supplemental Materials

**Supplementary Table 1. Total Urine Glycosaminoglycans Concentration (mg/mmol creatinine)**

| **Age** | **GAG Concentration** | **Mean** | **SD** | **3x Mean Normal Range** |
| --- | --- | --- | --- | --- |
| **0-5 months** | 14.6-47.8 | 29.9 | 8 | 89.7 |
| **6-11 months** | 3.7-35.5 | 19.6 | 6.3 | 58.8 |
| **1-2 years** | 5.4-30.8 | 17.8 | 5.2 | 53.4 |
| **3-6 years** | 5.2-16.7 | 10.9 | 2.4 | 32.7 |
| **7-13 years** | 2.4-10.2 | 8.0 | 2.4 | 24.0 |
| **≥14 years** | 0.0-7.1 | 4.7 | 2.0 | 14.1 |

Total urine glycosaminoglycans includes heparan sulfate, dermatan sulfate, and chondroitin sulfate.

**Supplementary Table 2. Domains for Heat Map Analysis**

| **Mobility** | **Fatigue** | **Fine Motor + Self-care** |
| --- | --- | --- |
| - Walking - Stairs - Balance - Running - Movement - Pain | - General fatigue - Sleep/rest fatigue - Cognitive fatigue | - Dexterity - Fine Motor Precision - Self-care - Dressing - Eating - Hygiene - Caregiver assistance |

**Supplemental Table 3. Individual Baseline Results for Assessments included in Heat Map Analysis**

| **Subject ID** | **Assessment** | **R** | **BL** | **Assessment** | **R** | **BL** | **Assessment** | **R** | **BL** |
| --- | --- | --- | --- | --- | --- | --- | --- | --- | --- |
| A4, F, 14.7 yr | 6MWT (m) | 300 | 300 | Total Fatigue | 46 | 46 | BOT2-Fine | 18 | 18 |
|  | 2MWT (m) | 82 | 82 | General Fatigue | 46 | 46 | BOT2-Dexterity | 12 | 12 |
|  | MPS HAQ-Walking* | 2 | 2 | Sleep/Rest Fatigue | 42 | 42 | CPE-Fine Motor* | 5 | 5 |
|  | 3MSC (Steps) | 86 | 86 | Cognitive Fatigue | 50 | 50 | CPE-Self Care* | 6 | 6 |
|  | MPS HAQ-Stairs* | 1 | 1 | CPE-Fatigue* | 5 | 5 | MPS HAQ-Dressing* | 5 | 5 |
|  | BOT2-Running | 4 | 4 |  |  |  | MPS HAQ-Eating & Drinking* | 3 | 3 |
|  | BOT2-Balance | 8 | 8 |  |  |  | MPS HAQ-Hygiene* | 4 | 4 |
|  | MPS HAQ-Movement* | 4 | 4 |  |  |  | MPS HAQ-Caregiver Assist* | 21 | 21 |
|  | PROMIS-Pain* | 25 | 25 |  |  |  |  |  |  |
| A11, F, 13.4 yr | 6MWT (m) | 19 | 19 | Total Fatigue | 50 | 50 | BOT2-Fine | 26 | 26 |
|  | 2MWT (m) | 16 | 16 | General Fatigue | 30 | 30 | BOT2-Dexterity | 13 | 13 |
|  | CPE-Walking* | 7 | 7 | Sleep/Rest Fatigue | 46 | 46 | MPS HAQ-Dressing* | 7 | 7 |
|  | MPS HAQ-Walking* | 10 | 10 | Cognitive Fatigue | 71 | 71 | MPS HAQ-Eating & Drinking* | 3 | 3 |
|  | MPS HAQ-Stairs* | 10 | 10 | CPE-Fatigue* | 7 | 7 | MPS HAQ-Hygiene* | 6 | 6 |
|  | MPS HAQ-Movement* | 8 | 8 |  |  |  | MPS HAQ-Caregiver Assist* | 29 | 29 |
|  | CHAQ-Pain* | 50 | 50 |  |  |  |  |  |  |
| A7, F, 11.5 yr | 6MWT (m) | 398 | 398 | Total Fatigue | 88 | 88 | BOT2-Fine | 25 | 25 |
|  | 2MWT (m) | 137 | 137 | General Fatigue | 96 | 96 | BOT2-Dexterity | 15 | 15 |
|  | MPS HAQ-Walking* | 0 | 0 | Sleep/Rest Fatigue | 100 | 100 | MPS HAQ-Dressing* | 0 | 0 |
|  | 3MSC (Steps) | 144 | 144 | Cognitive Fatigue | 67 | 67 | MPS HAQ-Eating & Drinking* | 0 | 0 |
|  | MPS HAQ-Stairs* | 0 | 0 |  |  |  | MPS HAQ-Hygiene* | 0 | 0 |
|  | BOT2-Running | 15 | 15 |  |  |  | MPS HAQ-Caregiver Assist* | 2 | 2 |
|  | CPE-Running* | 4 | 4 |  |  |  |  |  |  |
|  | BOT2-Balance | 15 | 15 |  |  |  |  |  |  |
|  | MPS HAQ-Movement* | 0 | 0 |  |  |  |  |  |  |
|  | CHAQ-Pain* | 10 | 10 |  |  |  |  |  |  |
| B8, F, 8.5 yr | 6MWT (m) | 200 | 188 | Total Fatigue | 58 | 71 | BOT2-Fine | 0 | 0 |
|  | 2MWT (m) | 89 | 80 | General Fatigue | 54 | 79 | BOT2-Dexterity | 1 | 1 |
|  | CPE-Walking* | 5 | 4 | Sleep/Rest Fatigue | 71 | 92 | MPS HAQ-Dressing* | 5 | 8 |
|  | MPS HAQ-Walking* | 1 | 1 | Cognitive Fatigue | 50 | 42 | MPS HAQ-Eating & Drinking* | 5 | 3 |
|  | 3MSC (Steps) | 59 | 59 | CPE-Fatigue* | 6 | 4 | MPS HAQ-Hygiene* | 7 | 7 |
|  | MPS HAQ-Stairs* | 3 | 6 |  |  |  | MPS HAQ-Caregiver Assist* | 23 | 26 |
|  | BOT2-Running | 0 | 0 |  |  |  |  |  |  |
|  | BOT2-Balance | 1 | 1 |  |  |  |  |  |  |
|  | MPS HAQ-Movement* | 6 | 6 |  |  |  |  |  |  |
|  | CHAQ-Pain* | 0 | 5 |  |  |  |  |  |  |
| B2, F, 12.8 yr | MPS HAQ-Walking* | 10 | 10 | Total Fatigue | 78 | 74 | BOT2-Fine | 1 | 0 |
|  | MPS HAQ-Stairs* | 10 | 10 | General Fatigue | 50 | 71 | BOT2-Dexterity | 0 | 0 |
|  | MPS HAQ-Movement* | 10 | 9 | Sleep/Rest Fatigue | 100 | 75 | CPE-Fine Motor* | 4 | 3 |
|  | CHAQ-Pain* | 40 | 25 | Cognitive Fatigue | 83 | 75 | MPS HAQ-Dressing* | 9 | 9 |
|  |  |  |  | CPE-Fatigue* | 6 | 4 | MPS HAQ-Eating & Drinking* | 3 | 4 |
|  |  |  |  |  |  |  | MPS HAQ-Hygiene* | 9 | 9 |
|  |  |  |  |  |  |  | MPS HAQ-Caregiver Assist* | 37 | 37 |
| B9, M, 16.5 yr | 6MWT (m) | 200 | 225 | Total Fatigue | 74 | 74 | BOT2-Fine | 24 | 30 |
|  | 2MWT (m) | 58 | 76 | General Fatigue | 83 | 75 | BOT2-Dexterity | 8 | 12 |
|  | CPE-Walking* | 6 | 5 | Sleep/Rest Fatigue | 75 | 83 | MPS HAQ-Dressing* | 1 | 1 |
|  | MPS HAQ-Walking* | 2 | 3 | Cognitive Fatigue | 63 | 63 | MPS HAQ-Eating & Drinking* | 1 | 0 |
|  | 3MSC (Steps) | 72 | 65 |  |  |  | MPS HAQ-Hygiene* | 1 | 2 |
|  | MPS HAQ-Stairs* | 4 | 3 |  |  |  | MPS HAQ-Caregiver Assist* | 10 | 7 |
|  | BOT2-Running | - | 1 |  |  |  |  |  |  |
|  | BOT2-Balance | 2 | 1 |  |  |  |  |  |  |
|  | MPS HAQ-Movement* | 2 | 2 |  |  |  |  |  |  |
|  | PROMIS-Pain* | 30 | 30 |  |  |  |  |  |  |

R refers to last non-missing value before or at randomization. BL refers to last non-missing value before or no the date of 1^st^ vestronidase alfa dose. BL, baseline; R, Randomization

**Supplemental Table 3 Continued**

| **Subject ID** | **Assessment** | **R** | **BL** | **Assessment** | **R** | **BL** | **Assessment** | **R** | **BL** |
| --- | --- | --- | --- | --- | --- | --- | --- | --- | --- |
| C1, F, 17.4 yr | CPE-Walking* | 6 | 4 | Total Fatigue | 61 | 63 | MPS HAQ-Dressing* | 8 | 8 |
|  | MPS HAQ-Walking* | 6 | 1 | General Fatigue | 71 | 83 | MPS HAQ-Eating & Drinking* | 4 | 2 |
|  | 3MSC (Steps) | 50 | 89 | Sleep/Rest Fatigue | 96 | 92 | MPS HAQ-Hygiene* | 7 | 6 |
|  | MPS HAQ-Stairs* | 8 | 0 | Cognitive Fatigue | 17 | 13 | MPS HAQ-Caregiver Assist* | 22 | 15 |
|  | CPE-Gross* | 6 | 4 |  |  |  |  |  |  |
|  | MPS HAQ-Movement* | 9 | 5 |  |  |  |  |  |  |
|  | PROMIS-Pain* | 70 | 40 |  |  |  |  |  |  |
| C5, F, 22.6 yr | 6MWT (m) | 511 | 569 | Total Fatigue | 25 | 43 | BOT2-Fine | 35 | 36 |
|  | 2MWT (m) | 160 | 195 | General Fatigue | 25 | 58 | BOT2-Dexterity | 34 | 34 |
|  | CPE-Walking* | 5 | 4 | Sleep/Rest Fatigue | 25 | 29 | MPS HAQ-Dressing* | 0 | 0 |
|  | MPS HAQ-Walking* | 1 | 1 | Cognitive Fatigue | 25 | 42 | MPS HAQ-Eating & Drinking* | 0 | 0 |
|  | 3MSC (Steps) | 208 | 174 | CPE-Fatigue* | 5 | 4 | MPS HAQ-Hygiene* | 0 | 0 |
|  | MPS HAQ-Stairs* | 0 | 1 |  |  |  | MPS HAQ-Caregiver Assist* | 0 | 0 |
|  | BOT2-Running | 21 | 25 |  |  |  |  |  |  |
|  | BOT2-Balance | 26 | 28 |  |  |  |  |  |  |
|  | MPS HAQ-Movement* | 1 | 0 |  |  |  |  |  |  |
|  | PROMIS-Pain* | 90 | 50 |  |  |  |  |  |  |
| C3, F, 22.5 yr | 6MWT (m) | 112 | 60 | Total Fatigue | 71 | 89 | BOT2-Fine | 24 | 24 |
|  | 2MWT (m) | 60 | 30 | General Fatigue | 79 | 92 | BOT2-Dexterity | 13 | 13 |
|  | CPE-Walking* | 6 | 6 | Sleep/Rest Fatigue | 50 | 75 | CPE-Self Care* | 5 | 2 |
|  | MPS HAQ-Walking* | 8 | 8 | Cognitive Fatigue | 83 | 100 | MPS HAQ-Dressing* | 3 | 2 |
|  | 3MSC (Steps) | 54 | 31 |  |  |  | MPS HAQ-Eating & Drinking* | 1 | 0 |
|  | CPE-Stairs* | 7 | 5 |  |  |  | MPS HAQ-Hygiene* | 2 | 2 |
|  | MPS HAQ-Stairs* | 8 | 8 |  |  |  | MPS HAQ-Caregiver Assist* | 19 | 12 |
|  | BOT2-Running | 2 | 1 |  |  |  |  |  |  |
|  | BOT2-Balance | 2 | 2 |  |  |  |  |  |  |
|  | MPS HAQ-Movement* | 3 | 4 |  |  |  |  |  |  |
|  | PROMIS-Pain* | 80 | 80 |  |  |  |  |  |  |
| D10, M, 10.1 yr | 6MWT (m) | 120 | 120 | Total Fatigue | 56 | 71 | BOT2-Fine | 11 | 16 |
|  | 2MWT (m) | 113 | 113 | General Fatigue | 58 | 63 | BOT2-Dexterity | 7 | 9 |
|  | MPS HAQ-Walking* | 7 | 5 | Sleep/Rest Fatigue | 79 | 88 | MPS HAQ-Dressing* | 7 | 4 |
|  | 3MSC (Steps) | 106 | 81 | Cognitive Fatigue | 29 | 63 | MPS HAQ-Eating & Drinking* | 3 | 2 |
|  | MPS HAQ-Stairs* | 0 | 3 | CPE-Fatigue* | 6 | 1 | MPS HAQ-Hygiene* | 4 | 3 |
|  | MPS HAQ-Movement* | 0 | 1 |  |  |  | MPS HAQ-Caregiver Assist* | 23 | 10 |
|  | CHAQ-Pain* | 50 | 45 |  |  |  |  |  |  |
| D6, M, 10.5 yr | CPE-Walking* | 7 | 7 | Total Fatigue | 67 | 58 | BOT2-Fine | 0 | 0 |
|  | MPS HAQ-Walking* | 10 | 10 | General Fatigue | 63 | 42 | BOT2-Dexterity | 1 | 1 |
|  | MPS HAQ-Stairs* | 10 | 10 | Sleep/Rest Fatigue | 100 | 100 | MPS HAQ-Dressing* | 10 | 10 |
|  | MPS HAQ-Movement* | 8 | 8 | Cognitive Fatigue | 38 | 33 | MPS HAQ-Eating & Drinking* | 5 | 5 |
|  | CHAQ-Pain* | 50 | 1 |  |  |  | MPS HAQ-Hygiene* | 8 | 9 |
|  |  |  |  |  |  |  | MPS HAQ-Caregiver Assist* | 37 | 37 |
| D12, M, 25.3 yr | 6MWT (m) | 506 | 455 | Total Fatigue | 43 | 47 | BOT2-Fine | 34 | 37 |
|  | 2MWT (m) | 176 | 157 | General Fatigue | 83 | 50 | BOT2-Dexterity | 19 | 20 |
|  | CPE-Walking* | 4 | 2 | Sleep/Rest Fatigue | 4 | 42 | MPS HAQ-Dressing* | 0 | 0 |
|  | MPS HAQ-Walking* | 0 | 4 | Cognitive Fatigue | 42 | 50 | MPS HAQ-Eating & Drinking* | 0 | 0 |
|  | 3MSC (Steps) | 216 | 173 |  |  |  | MPS HAQ-Hygiene* | 0 | 0 |
|  | MPS HAQ-Stairs* | 0 | 2 |  |  |  | MPS HAQ-Caregiver Assist* | 0 | 0 |
|  | BOT2-Running | 31 | 18 |  |  |  |  |  |  |
|  | CPE-Running* | 3 | 1 |  |  |  |  |  |  |
|  | BOT2-Balance | 28 | 27 |  |  |  |  |  |  |
|  | CPE-Gross* | 4 | 1 |  |  |  |  |  |  |
|  | MPS HAQ-Movement* | 0 | 1 |  |  |  |  |  |  |
|  | PROMIS-Pain* | 75 | 50 |  |  |  |  |  |  |

R refers to last non-missing value before or at randomization. BL refers to last non-missing value before or no the date of 1^st^ vestronidase alfa dose. BL, baseline; R, Randomization

**Supplementary Table 4. List of Preferred Terms Included in Surgical and Medical Procedures**

| **Surgical & Medical Procedures** |
| --- |
| - Adenoidectomy - Appendicectomy - Cast application - Cholecystectomy - Circumcision - Ear tube insertion - Inguinal hernia repair - Medical device implantation - Medical device removal - Myringotomy - Osteotomy - Spinal decompression - Spinal fusion surgery - Spinal laminectomy - Tenotomy - Tonsillectomy - Tooth extraction - Umbilical hernia repair |

**Supplementary Figure 1. Individual Heat Map Response With Numbers**

|  | | **Mobility** | | | | | | | | | | | | | | | **Fatigue** | | | | | | | | | | | | | **Fine-Motor & Self-Care** | | | | | | | | | | | | | | | | | | |
| --- | --- | --- | --- | --- | --- | --- | --- | --- | --- | --- | --- | --- | --- | --- | --- | --- | --- | --- | --- | --- | --- | --- | --- | --- | --- | --- | --- | --- | --- | --- | --- | --- | --- | --- | --- | --- | --- | --- | --- | --- | --- | --- | --- | --- | --- | --- | --- | --- |
|  | | **Week** | **8** | | | **16** | | | **24** | | **32** | | **40** | | **48** | | **Week** | | **8** | | **16** | | **24** | | **32** | | **40** | | **48** | **Week** | | **8** | | **16** | | | **24** | | | **32** | | | **40** | | | **48** | | |
| **A4**  **Female 14.7-yrs** | | 6MWT (m) | 20 | | | 80 | | | 83 | | 84 | | 82 | | 90 | | Total Fatigue | | 5 | | -7 | | 5 | | 22 | | 18 | | 18 | BOT2-Fine | | -3 | | 3 | | | 2 | | | 1 | | | 1 | | | -3 | | |
|  |  | 2MWT (m) | 58 | | | 60 | | | 58 | | 58 | | 61 | | 66 | | General Fatigue | | -4 | | -29 | | 4 | | 25 | | 8 | | 8 | BOT2-Dexterity | | 0 | | -3 | | | -1 | | | 1 | | | 0 | | | -2 | | |
|  |  | MPS HAQ-Walking* | -2 | | | -1 | | | -1 | | -2 | | 0 | | 0 | | Sleep/Rest Fatigue | | 21 | | 8 | | 12 | | 29 | | 33 | | 33 | CPE-Fine Motor* | | 0 | | 0 | | | 0 | | | 0 | | | 0 | | | 1 | | |
|  |  | 3MSC (Steps) | 12 | | | 12 | | | 11 | | 1 | | 1 | | -8 | | Cognitive Fatigue | | 0 | | 0 | | 0 | | 13 | | 13 | | 13 | CPE-Self Care* | | 0 | | 0 | | | 0 | | | 0 | | | 0 | | | 0 | | |
|  |  | MPS HAQ-Stairs* | -2 | | | -3 | | | -2 | | -2 | | -1 | | -3 | | CPE-Fatigue | | 0 | | -1 | | 0 | | 0 | | 0 | | 1 | MPS HAQ-Dressing* | | 1 | | 0 | | | 0 | | | 2 | | | 2 | | | 3 | | |
|  |  | BOT2-Running | 0 | | | 0 | | | 0 | | 0 | | -1 | |  | |  | |  | |  | |  | |  | |  | |  | MPS HAQ-Eating & Drinking* | | 1 | | 1 | | | 2 | | | 2 | | | 2 | | | 1 | | |
|  |  | BOT2-Balance | -2 | | | 1 | | | 4 | | 5 | | 5 | | 6 | |  | |  | |  | |  | |  | |  | |  | MPS HAQ-Hygiene* | | 0 | | 0 | | | 0 | | | 0 | | | 1 | | | 1 | | |
|  |  | MPS HAQ-Movement* | 0 | | | 1 | | | 2 | | 1 | | 1 | | 1 | |  | |  | |  | |  | |  | |  | |  | MPS HAQ-Caregiver Assist* | | 7 | | -8 | | | -5 | | | 3 | | | 6 | | | 2 | | |
|  |  | PROMIS-Pain* | -50 | | | -70 | | | -25 | | 0 | | 5 | | 5 | |  | |  | |  | |  | |  | |  | |  |  | |  | |  | | |  | | |  | | |  | | |  | | |
|  | |  |  | | |  | | |  | |  | |  | |  | |  | |  | |  | |  | |  | |  | |  |  | |  | |  | | |  | | |  | | |  | | |  | | |
| **A11**  **Female 13.4-yrs** | | 6MWT (m) | 3 | | | 3 | | | 4 | | 4 | | 4 | | 1 | | Total Fatigue | | 12 | | 12 | | -6 | | -8 | | -7 | | 6 | BOT2-Fine | | -1 | | 3 | | | 5 | | | 7 | | | 1 | | | 5 | | |
|  |  | 2MWT (m) | -8 | | | -9 | | | -9 | | -8 | | -8 | | -9 | | General Fatigue | | 5 | | 20 | | 12 | | 8 | | -1 | | 3 | BOT2-Dexterity | | 2 | | 2 | | | 3 | | | 3 | | | 3 | | | 6 | | |
|  |  | CPE-Walking* | 0 | | | 0 | | | 0 | | 0 | | 0 | | 0 | | Sleep/Rest Fatigue | | 8 | | 29 | | -8 | | -17 | | -4 | | 21 | MPS HAQ-Dressing* | | 2 | | 0 | | | 0 | | | 0 | | | 0 | | | -1 | | |
|  |  | MPS HAQ-Walking* | 1 | | | 0 | | | 0 | | 0 | | 0 | | 0 | | Cognitive Fatigue | | 21 | | -8 | | -17 | | -13 | | -13 | | -4 | MPS HAQ-Eating & Drinking* | | 2 | | 1 | | | 1 | | | -1 | | | 2 | | | 1 | | |
|  |  | MPS HAQ-Stairs* | 0 | | | 0 | | | 0 | | 0 | | 0 | | 0 | | CPE-Fatigue | | 1 | | 1 | | 1 | | 1 | | 2 | | 1 | MPS HAQ-Hygiene* | | 2 | | 0 | | | 0 | | | 0 | | | 1 | | | 1 | | |
|  |  | MPS HAQ-Movement* | -1 | | | 1 | | | 2 | | 0 | | 1 | | 2 | |  | |  | |  | |  | |  | |  | |  | MPS HAQ-Caregiver Assist* | | -3 | | 5 | | | 8 | | | -1 | | | 1 | | | 1 | | |
|  |  | CHAQ-Pain* | 10 | | | -30 | | | 0 | | -16 | | 0 | | -25 | |  | |  | |  | |  | |  | |  | |  |  | |  | |  | | |  | | |  | | |  | | |  | | |
|  | |  |  | | |  | | |  | |  | |  | |  | |  | |  | |  | |  | |  | |  | |  |  | |  | |  | | |  | | |  | | |  | | |  | | |
| **A7**  **Female 11.5-yrs** | | 6MWT (m) | 2 | | | 22 | | | -29 | | 12 | | 11 | | -44 | | Total Fatigue | | -10 | | 1 | | 11 | | 5 | | 6 | | -3 | BOT2-Fine | | 0 | | -5 | | | -1 | | | -4 | | | -3 | | | -2 | | |
|  |  | 2MWT (m) | -1 | | | 5 | | | -11 | | 5 | | 3 | | -14 | | General Fatigue | | -8 | | -4 | | 4 | | 0 | | -4 | | -4 | BOT2-Dexterity | | -4 | | -4 | | | -2 | | | -2 | | | -2 | | | -1 | | |
|  |  | MPS HAQ-Walking* | 0 | | | 0 | | | 0 | | 0 | | 0 | | 0 | | Sleep/Rest Fatigue | | 0 | | 0 | | 0 | | 0 | | 0 | | 0 | MPS HAQ-Dressing* | | 0 | | 0 | | | 0 | | | 0 | | | 0 | | | 0 | | |
|  |  | 3MSC (Steps) | -16 | | | -18 | | | -54 | | -43 | | -28 | | -40 | | Cognitive Fatigue | | -21 | | 8 | | 29 | | 16 | | 25 | | -4 | MPS HAQ-Eating & Drinking* | | 0 | | -1 | | | 0 | | | 0 | | | 0 | | | 0 | | |
|  |  | MPS HAQ-Stairs* | 0 | | | 0 | | | 0 | | 0 | | 0 | | 0 | |  | |  | |  | |  | |  | |  | |  | MPS HAQ-Hygiene* | | 0 | | 0 | | | 0 | | | 0 | | | 0 | | | 0 | | |
|  |  | BOT2-Running | -6 | | | 5 | | | -1 | | -4 | | -4 | | -7 | |  | |  | |  | |  | |  | |  | |  | MPS HAQ-Caregiver Assist* | | 0 | | 2 | | | 1 | | | 1 | | | 1 | | | 1 | | |
|  |  | CPE-Running* | -1 | | | 1 | | | 1 | | 1 | | 3 | | 3 | |  | |  | |  | |  | |  | |  | |  |  | |  | |  | | |  | | |  | | |  | | |  | | |
|  |  | BOT2-Balance | -1 | | | 1 | | | 4 | | -1 | | -3 | | 5 | |  | |  | |  | |  | |  | |  | |  |  | |  | |  | | |  | | |  | | |  | | |  | | |
|  |  | MPS HAQ-Movement* | -1 | | | 0 | | | 0 | | 0 | | 0 | | 0 | |  | |  | |  | |  | |  | |  | |  |  | |  | |  | | |  | | |  | | |  | | |  | | |
|  |  | CHAQ-Pain* | 0 | | | 10 | | | 10 | | 7 | | 10 | | 5 | |  | |  | |  | |  | |  | |  | |  |  | |  | |  | | |  | | |  | | |  | | |  | | |
|  |  | | | | | | | | | | | | | | |  | | | | | | | | | | | | | |  | | | | | | | | | | | | | | | | | | |
| **B8**  **Female  8.5-yrs** | 6MWT (m) | | | -12 | | |  | | 21 | |  | |  | |  | | Total Fatigue | 13 | | 5 | | 14 | | 20 | | 19 | | 10 | | BOT2-Fine | 0 | |  | | | 1 | | |  | | |  | | |  | | |  |
|  | 2MWT (m) | | | -9 | | |  | | 0 | |  | |  | |  | | General Fatigue | 25 | | 13 | | 4 | | 9 | | 13 | | 9 | | BOT2-Dexterity | 0 | | -1 | | | 0 | | | -1 | | | -1 | | | 0 | | |  |
|  | CPE-Walking* | | | 1 | | | 3 | | 3 | | 3 | | 3 | | 3 | | Sleep/Rest Fatigue | 21 | | 0 | | 8 | | 0 | | 8 | | -21 | | MPS HAQ-Dressing* | -3 | | 3 | | | 3 | | | 5 | | | 6 | | | 4 | | |  |
|  | MPS HAQ-Walking* | | | 0 | | | 0 | | 1 | | 1 | | 1 | | 1 | | Cognitive Fatigue | -8 | | 4 | | 28 | | 52 | | 37 | | 43 | | MPS HAQ-Eating & Drinking* | 2 | | 0 | | | -2 | | | 2 | | | 1 | | | -1 | | |  |
|  | 3MSC (Steps) | | | -32 | | | -8 | |  | |  | |  | |  | | CPE-Fatigue | 2 | | 3 | | 3 | | 3 | | 3 | | 3 | | MPS HAQ-Hygiene* | 0 | | 1 | | | 1 | | | 2 | | | 3 | | | 1 | | |  |
|  | MPS HAQ-Stairs* | | | -3 | | | -1 | | 1 | | 3 | | 6 | | 0 | |  |  | |  | |  | |  | |  | |  | | MPS HAQ-Caregiver Assist* | -3 | | -2 | | | 11 | | | 5 | | | 10 | | | 9 | | |  |
|  | MPS HAQ-Movement* | | | 0 | | | -1 | | 1 | | 1 | | 2 | | 2 | |  |  | |  | |  | |  | |  | |  | |  |  | |  | | |  | | |  | | |  | | |  | | |  |
|  | CHAQ-Pain* | | | -5 | | | 5 | | 5 | | 5 | | 5 | | 5 | |  |  | |  | |  | |  | |  | |  | |  |  | |  | | |  | | |  | | |  | | |  | | |  |
|  |  | | |  |  | | |  | |  | |  | |  | |  | |  | |  | |  | |  | |  | |  | |  |  | |  | | |  | | |  | | |  | | |  | | |  |
| **B2**  **Female 12.8-yrs** | MPS HAQ-Walking* | | | 0 | | | 0 | | 0 | | 0 | | 0 | | 0 | Total Fatigue | | -4 | | 14 | | -3 | | 7 | | 18 | | 4 | | BOT2-Fine | -1 | | 0 | | | 0 | | | 0 | | |  | | | 0 | | |  |
|  | MPS HAQ-Stairs* | | | 0 | | | 0 | | 0 | | 0 | | 0 | | 0 | General Fatigue | | 21 | | 0 | | -4 | | 8 | | 8 | | -8 | | BOT2-Dexterity | 0 | | 0 | | | 0 | | | 0 | | | 0 | | | 0 | | |  |
|  | MPS HAQ-Movement* | | | 1 | | | 0 | | 0 | | 1 | | -1 | | -1 | Sleep/Rest Fatigue | | -25 | | 21 | | -4 | | 8 | | 21 | | 8 | | CPE-Fine Motor* | 1 | | -1 | | | 2 | | | 1 | | | 2 | | | 2 | | |  |
|  | CHAQ-Pain* | | | 15 | | | 5 | | 25 | | -35 | | -5 | | 5 | Cognitive Fatigue | | -8 | | 21 | | 0 | |  | | 25 | | 13 | | MPS HAQ-Dressing* | 0 | | 1 | | | -1 | | | -1 | | | -1 | | | -1 | | |  |
|  |  | | |  | | |  | |  | |  | |  | |  | CPE-Fatigue | | 2 | | 1 | | 0 | | 1 | | 1 | | 0 | | MPS HAQ-Eating & Drinking* | -1 | | 0 | | | -1 | | | -1 | | | -1 | | | 2 | | |  |
|  |  | | |  | | |  | |  | |  | |  | |  |  | |  | |  | |  | |  | |  | |  | | MPS HAQ-Hygiene* | 0 | | 0 | | | 0 | | | 0 | | | -1 | | | 0 | | |  |
|  |  | | |  | | |  | |  | |  | |  | |  |  | |  | |  | |  | |  | |  | |  | | MPS HAQ-Caregiver Assist* | 0 | | -2 | | | -1 | | | 1 | | | 0 | | | -1 | | |  |
|  |  | | |  | | |  | |  | |  | |  | |  |  | |  | |  | |  | |  | |  | |  | |  |  | |  | | |  | | |  | | |  | | |  | | |  |
| **B9**  **Male  16.5-yrs** | 6MWT (m) | | | 25 | | | -9 | | -14 | | 3 | | -22 | | 5 | Total Fatigue | | 0 | | 1 | | 15 | | 18 | | 16 | | 4 | | BOT2-Fine | 6 | | -3 | | | -2 | | | -7 | | | -2 | | | 1 | | |  |
|  | 2MWT (m) | | | 18 | | | 2 | | 1 | | 3 | | -4 | | 0 | General Fatigue | | -8 | | 8 | | 8 | | 21 | | 21 | | 8 | | BOT2-Dexterity | 4 | | 2 | | | 0 | | | 2 | | | 1 | | | 0 | | |  |
|  | CPE-Walking* | | | 1 | | | 1 | | 1 | | -1 | | 0 | | 0 | Sleep/Rest Fatigue | | 8 | | -4 | | 17 | | 13 | | 13 | | 5 | | MPS HAQ-Dressing* | 0 | | -1 | | | 0 | | | 0 | | | 0 | | | -1 | | |  |
|  | MPS HAQ-Walking* | | | -1 | | | 0 | | 2 | | 2 | | 1 | | 2 | Cognitive Fatigue | | 0 | | 0 | | 20 | | 20 | | 16 | | 0 | | MPS HAQ-Eating & Drinking* | 1 | | -1 | | | 0 | | | 0 | | | 0 | | | 0 | | |  |
|  | 3MSC (Steps) | | | -7 | | | 0 | | 15 | | 11 | | 12 | | 15 |  | |  | | |  | |  | |  | |  | |  | MPS HAQ-Hygiene* | -1 | | 0 | | | 2 | | | 1 | | | 1 | | | 1 | | |  |
|  | MPS HAQ-Stairs* | | | 1 | | | 0 | | 3 | | 2 | | 2 | | 2 |  | |  | | |  | |  | |  | |  | |  | MPS HAQ-Caregiver Assist* | 3 | | 3 | | | 2 | | | 2 | | | 2 | | | 0 | | |  |
|  | BOT2-Running | | |  | | | 1 | | 2 | | 1 | | 1 | | 2 |  | |  | | |  | |  | | \|  \|  \|  \|  \|  \|  \|  \| \| --- \| --- \| --- \| --- \| --- \| --- \| --- \|   Worsening Improving  Cells right of the green line- vestronidase alfa; Cells left of the green line – placebo | |  | |  |  |  | | | |  | | |  | | |  | | |  | | |  |  |
|  | BOT2-Balance | | | -1 | | | 13 | | 4 | | 3 | | 12 | | 6 |  | |  | | |  | |  | |  | |  | |  |  |  | | | |  | | |  | | |  | | |  | | |  |  |
|  | MPS HAQ-Movement* | | | 0 | | | 0 | | 1 | | 1 | | 1 | | 1 |  | |  | | |  | |  | |  | |  | |  |  |  | | | |  | | |  | | |  | | |  | | |  |  |
|  | PROMIS-Pain* | | | 0 | | | 0 | | 20 | | -10 | | 10 | | 10 |  | |  | | |  | |  | |  | |  | |  |  |  | | | |  | | |  | | |  | | |  | | |  |  |

**Supplemental Figure 1. Individual Heat Map Response with Numbers (Continued)**

|  | | **Mobility** | | | | | | | | | | | | | | | | | | **Fatigue** | | | | | | | | | | | | | | | | | | | | | | | | | | | | | | | | **Fine-Motor & Self Care** | | | | | | | | | | | | | | | | | | | | | | | | | | | | | | |  |
| --- | --- | --- | --- | --- | --- | --- | --- | --- | --- | --- | --- | --- | --- | --- | --- | --- | --- | --- | --- | --- | --- | --- | --- | --- | --- | --- | --- | --- | --- | --- | --- | --- | --- | --- | --- | --- | --- | --- | --- | --- | --- | --- | --- | --- | --- | --- | --- | --- | --- | --- | --- | --- | --- | --- | --- | --- | --- | --- | --- | --- | --- | --- | --- | --- | --- | --- | --- | --- | --- | --- | --- | --- | --- | --- | --- | --- | --- | --- | --- | --- | --- | --- | --- |
|  | | **Week** | | **8** | | **16** | | | **24** | | | **32** | | | **40** | | | **48** | | **Week** | | | | **8** | | | **16** | | | | | **24** | | | | | | **32** | | | | | | **40** | | | | | | **48** | | **Week** | | | | **8** | | | | **16** | | | | | **24** | | | | | **32** | | | | | **40** | | | | | **48** | | |  |
| **C1**  **Female  17.4-yrs** | | CPE-Walking* | | 0 | | 2 | | | -1 | | | -1 | | | 2 | | | 0 | | Total Fatigue | | | | -1 | | | 2 | | | | | 10 | | | | | | 23 | | | | | | -3 | | | | | | 7 | | MPS HAQ-Dressing* | | | | 1 | | | | 0 | | | | | -1 | | | | | -1 | | | | | 0 | | | | | -1 | | |  |
|  |  | MPS HAQ-Walking* | | 2 | | 5 | | | -5 | | | -2 | | | -2 | | | -2 | | General Fatigue | | | | 12 | | | 12 | | | | | -16 | | | | | | 9 | | | | | | 5 | | | | | | -4 | | MPS HAQ-Eating & Drinking* | | | | 2 | | | | 2 | | | | | -1 | | | | | -1 | | | | | 0 | | | | | -1 | | |  |
|  |  | 3MSC (Steps) | | 35 | | 39 | | | 3 | | | -16 | | |  | | | -25 | | Sleep/Rest Fatigue | | | | -25 | | | -4 | | | | | 0 | | | | | | 4 | | | | | | -17 | | | | | | -4 | | MPS HAQ-Hygiene* | | | | 0 | | | | 1 | | | | | -1 | | | | | -1 | | | | | -1 | | | | | -1 | | |  |
|  |  | MPS HAQ-Stairs* | | 4 | | 8 | | | -4 | | | -1 | | | -2 | | | -1 | | Cognitive Fatigue | | | | 8 | | | -4 | | | | | 43 | | | | | | 50 | | | | | | -3 | | | | | | 4 | | MPS HAQ-Caregiver Assist* | | | | -1 | | | | 7 | | | | | -10 | | | | | -7 | | | | | -5 | | | | | -9 | | |  |
|  |  | CPE-Gross* | | 0 | | 2 | | |  | | | -2 | | | 0 | | | 1 | |  | | | |  | | |  | | | | |  | | | | | |  | | | | | |  | | | | | |  | |  | | | |  | | | |  | | | | |  | | | | |  | | | | |  | | | | |  | | |  |
|  |  | MPS HAQ-Movement* | | 1 | | 4 | | | -2 | | | -2 | | | -2 | | | -3 | |  | | | |  | | |  | | | | |  | | | | | |  | | | | | |  | | | | | |  | |  | | | |  | | | |  | | | | |  | | | | |  | | | | |  | | | | |  | | |  |
|  |  | PROMIS-Pain* | | 70 | | 30 | | | 20 | | |  | | | -10 | | | 30 | |  | | | |  | | |  | | | | |  | | | | | |  | | | | | |  | | | | | |  | |  | | | |  | | | |  | | | | |  | | | | |  | | | | |  | | | | |  | | |  |
|  | |  | |  | |  | | |  | | |  | | |  | | |  | |  | | | |  | | |  | | | | |  | | | | | |  | | | | | |  | | | | | |  | |  | | | |  | | | |  | | | | |  | | | | |  | | | | |  | | | | |  | | |  |
| **C5**  **Female 22.6-yrs** | | 6MWT (m) | | 29 | | 58 | | | -49 | | | -31 | | |  | | | -2 | | Total Fatigue | | | | 32 | | | 18 | | | | | 4 | | | | | | -4 | | | | | | -10 | | | | | | 22 | | BOT2-Fine | | | | 1 | | | | 1 | | | | | 2 | | | | | 1 | | | | |  | | | | | 3 | | |  |
|  |  | 2MWT (m) | | 21 | | 35 | | | -10 | | | -13 | | |  | | | -1 | | General Fatigue | | | | 42 | | | 33 | | | | | -8 | | | | | | -16 | | | | | | -33 | | | | | | 13 | | BOT2-Dexterity | | | | 0 | | | | 0 | | | | | 1 | | | | | 1 | | | | |  | | | | | 1 | | |  |
|  |  | CPE-Walking* | | 4 | | 1 | | | 2 | | | 3 | | | -1 | | | 0 | | Sleep/Rest Fatigue | | | | 17 | | | 4 | | | | | 13 | | | | | | -4 | | | | | | -4 | | | | | | 25 | | MPS HAQ-Dressing* | | | | 0 | | | | 0 | | | | | 0 | | | | | 0 | | | | | 0 | | | | | 0 | | |  |
|  |  | MPS HAQ-Walking* | | 0 | | 0 | | | -3 | | | -4 | | | -1 | | | -2 | | Cognitive Fatigue | | | | 38 | | | 17 | | | | | 8 | | | | | | 8 | | | | | | 8 | | | | | | 29 | | MPS HAQ-Eating & Drinking* | | | | 0 | | | | 0 | | | | | 0 | | | | | 0 | | | | | 0 | | | | | 0 | | |  |
|  |  | 3MSC (Steps) | | -4 | | -34 | | | 20 | | | 25 | | |  | | | 49 | | CPE-Fatigue | | | | 1 | | | 1 | | | | | 1 | | | | | | 0 | | | | | | -1 | | | | | | 0 | | MPS HAQ-Hygiene* | | | | 0 | | | | 0 | | | | | 0 | | | | | 0 | | | | | 0 | | | | | 0 | | |  |
|  |  | MPS HAQ-Stairs* | | 0 | | -1 | | | -3 | | | -1 | | | 0 | | | -2 | |  | | | |  | | |  | | | | |  | | | | | |  | | | | | |  | | | | | |  | | MPS HAQ-Caregiver Assist* | | | | 0 | | | | 0 | | | | | 0 | | | | | 0 | | | | | 0 | | | | | 0 | | |  |
|  |  | BOT2-Running | |  | | 4 | | |  | | |  | | |  | | |  | |  | | | |  | | |  | | | | |  | | | | | |  | | | | | |  | | | | | |  | |  | | | |  | | | |  | | | | |  | | | | |  | | | | |  | | | | |  | | |  |
|  |  | BOT2-Balance | | 4 | | 2 | | | -1 | | | -1 | | |  | | |  | |  | | | |  | | |  | | | | |  | | | | | |  | | | | | |  | | | | | |  | |  | | | |  | | | |  | | | | |  | | | | |  | | | | |  | | | | |  | | |  |
|  |  | MPS HAQ-Movement* | | 1 | | 1 | | | -1 | | | -1 | | | 0 | | | -1 | |  | | | |  | | |  | | | | |  | | | | | |  | | | | | |  | | | | | |  | |  | | | |  | | | |  | | | | |  | | | | |  | | | | |  | | | | |  | | |  |
|  |  | PROMIS-Pain* | | 15 | | 40 | | | -25 | | | -25 | | | -40 | | | -50 | |  | | | |  | | |  | | | | |  | | | | | |  | | | | | |  | | | | | |  | |  | | | |  | | | |  | | | | |  | | | | |  | | | | |  | | | | |  | | |  |
|  | |  | |  | |  | | |  | | |  | | |  | | |  | |  | | | |  | | |  | | | | |  | | | | | |  | | | | | |  | | | | | |  | |  | | | |  | | | |  | | | | |  | | | | |  | | | | |  | | | | |  | | |  |
| **C3**  **Female  22.5-yrs** | | 6MWT (m) | | 28 | | -52 | | | 0 | | | 105 | | | 80 | | | 65 | | Total Fatigue | | | | 17 | | | 18 | | | | | -7 | | | | | | -4 | | | | | | 0 | | | | | | -6 | | BOT2-Fine | | | | 3 | | | | 0 | | | | | 1 | | | | | -1 | | | | | -3 | | | | | -5 | | |  |
|  |  | 2MWT (m) | | -17 | | -30 | | | -10 | | | 30 | | | 20 | | | 10 | | General Fatigue | | | | 17 | | | 13 | | | | | -17 | | | | | | 4 | | | | | | 4 | | | | | | 0 | | BOT2-Dexterity | | | | -1 | | | | 0 | | | | | 3 | | | | | 0 | | | | | 3 | | | | | 3 | | |  |
|  |  | CPE-Walking* | | 2 | | 0 | | | 0 | | | 5 | | | 1 | | | 1 | | Sleep/Rest Fatigue | | | | 29 | | | 25 | | | | | 13 | | | | | | -4 | | | | | | 0 | | | | | | 4 | | CPE-Self Care* | | | | 3 | | | | 3 | | | | | -1 | | | | | 1 | | | | | 1 | | | | | 1 | | |  |
|  |  | MPS HAQ-Walking* | | 0 | | 0 | | | -1 | | | 3 | | | -1 | | | 1 | | Cognitive Fatigue | | | | 5 | | | 17 | | | | | -17 | | | | | | -12 | | | | | | -4 | | | | | | -21 | | MPS HAQ-Dressing* | | | | 0 | | | | 1 | | | | | 0 | | | | | 0 | | | | | 1 | | | | | 1 | | |  |
|  |  | 3MSC (Steps) | | 4 | | -23 | | | 14 | | | 17 | | | -1 | | | 0 | |  | | | |  | |  | | | |  | | | | | |  | | | | | | |  | | | | | |  | | | MPS HAQ-Eating & Drinking* | | | | 0 | | | | 1 | | | | | 0 | | | | | -1 | | | | | 0 | | | | | 0 | | |  |
|  |  | CPE-Stairs* | | 2 | | 2 | | | 2 | | | 0 | | | 3 | | | 0 | |  | | | |  | |  | | | |  | | | | | |  | | | | | | |  | | | | | |  | | | MPS HAQ-Hygiene* | | | | 0 | | | | 0 | | | | | 0 | | | | | 1 | | | | | 0 | | | | | 1 | | |  |
|  |  | MPS HAQ-Stairs* | | 2 | | 0 | | | 1 | | | 1 | | | 0 | | | 3 | |  | | | |  |  | | |  | | | | | |  | | | | | |  | | | | | |  | | | | | | MPS HAQ-Caregiver Assist* | | | | 14 | | | | 7 | | | | | 0 | | | | | 5 | | | | | -1 | | | | | 6 | | |  |
|  |  | BOT2-Running | | 0 | | -1 | | | -1 | | | 0 | | | 0 | | | 0 | |  | | | |  |  | | |  | | | | | |  | | | | | |  | | | | | |  | | | | | |  | |  | | | |  | | | | |  | | | | |  | | | | |  | | | | |  | | | | |  |
|  |  | BOT2-Balance | | 0 | | 0 | | | 7 | | | 7 | | | 0 | | | 6 | |  | | | |  |  | | |  | | | | | |  | | | | | |  | | | | | |  | | | | | |  | |  | | | |  | | | | |  | | | | |  | | | | |  | | | | |  | | | | |  |
|  |  | MPS HAQ-Movement* | | -1 | | -1 | | | 0 | | | 1 | | | 1 | | | 1 | |  | | | |  |  | | |  | | | | | |  | | | | | |  | | | | | |  | | | | | |  | |  | | | |  | | | | |  | | | | |  | | | | |  | | | | |  | | | | |  |
|  |  | PROMIS-Pain* | | 50 | | 0 | | | 10 | | | 50 | | | 70 | | | 80 | |  | | | |  |  | | |  | | | | | |  | | | | | |  | | | | | |  | | | | | |  | |  | | | |  | | | | |  | | | | |  | | | | |  | | | | |  | | | | |  |
|  | |  | |  | | | |  |  | |  | | |  | | |  | | |  | |  | | | |  | | |  | | | | | |  | | | | | |  | | | | | |  | | | | |  | | |  | | | |  | | | | |  | | | | |  | | | | |  | | | | |  | | | | |
| **D10**  **Male 10.1-yrs** | | 6MWT (m) | |  | |  | | |  | | | -49 | | |  | | |  | | Total Fatigue | | -6 | | | | 5 | | | | | | 15 | | | | | | 4 | | | | | | 4 | | | | | | -3 | | BOT2-Fine | | | | 4 | | | | 4 | | | | | 5 | | | | | 0 | | | | | -7 | | | | | 1 | | | |
|  |  | 2MWT (m) | |  | |  | | |  | | | -42 | | |  | | |  | | General Fatigue | | -12 | | | | -4 | | | | | | 5 | | | | | | 8 | | | | | | 12 | | | | | | 0 | | BOT2-Dexterity | | | | 1 | | | | -1 | | | | | 2 | | | | | -3 | | | | | 0 | | | | | 1 | | | |
|  |  | MPS HAQ-Walking* | | 1 | | 1 | | | 2 | | | -2 | | | 0 | | | -1 | | Sleep/Rest Fatigue | | -4 | | | | 0 | | | | | | 9 | | | | | | -5 | | | | | | 4 | | | | | | 4 | | MPS HAQ-Dressing* | | | | 2 | | | | 2 | | | | | 3 | | | | | 0 | | | | | 0 | | | | | 0 | | | |
|  |  | 3MSC (Steps) | | -17 | | -20 | | | -25 | | | -30 | | |  | | | -32 | | Cognitive Fatigue | | 0 | | | | 21 | | | | | | 34 | | | | | | 8 | | | | | | -5 | | | | | | -13 | | MPS HAQ-Eating & Drinking* | | | | 1 | | | | 2 | | | | | 1 | | | | | 1 | | | | | 1 | | | | | 1 | | | |
|  |  | MPS HAQ-Stairs* | | -5 | | -5 | | | -3 | | | -2 | | | -2 | | | -2 | | CPE-Fatigue | | 4 | | | | 5 | | | | | | 5 | | | | | | 0 | | | | | | 0 | | | | | | 0 | | MPS HAQ-Hygiene* | | | | 0 | | | | 0 | | | | | 1 | | | | | 1 | | | | | 0 | | | | | 0 | | | |
|  |  | MPS HAQ-Movement* | | -1 | | -1 | | | -1 | | | 1 | | | 0 | | | 0 | |  | |  | | | |  | | | | | |  | | | | | |  | | | | | |  | | | | | |  | | MPS HAQ-Caregiver Assist* | | | | 0 | | | | 6 | | | | | 13 | | | | | -3 | | | | | -1 | | | | | -1 | | | |
|  |  | CHAQ-Pain* | | 0 | | 0 | | | 5 | | | 0 | | | 0 | | | 0 | |  | |  | | | |  | | | | | |  | | | | | |  | | | | | |  | | | | | |  | |  | | | |  | | | |  | | | | |  | | | | |  | | | | |  | | | | |  | | | |
|  | |  | |  | |  | | |  | | |  | | |  | | |  | |  | |  | | | |  | | | | | |  | | | | | |  | | | | | |  | | | | | |  | |  | | | |  | | | |  | | | | |  | | | | |  | | | | |  | | | | |  | | | |
| **D6**  **Male  10.5-yrs** | | CPE-Walking* | | 0 | | 0 | | | 0 | | | 0 | | | 0 | | | 1 | | Total Fatigue | | -2 | | | | -18 | | | | | | -9 | | | | | | 10 | | | | | | 17 | | | | | | 10 | | BOT2-Fine | | | | 0 | | | | 0 | | | | | 0 | | | | | 0 | | | | | 0 | | | | | 0 | | | |
|  |  | MPS HAQ-Walking* | | 0 | | 0 | | | 0 | | | 0 | | | 0 | | | 0 | | General Fatigue | | 4 | | | | -17 | | | | | | -21 | | | | | | 12 | | | | | | 37 | | | | | | 12 | | BOT2-Dexterity | | | | 2 | | | | 2 | | | | | 0 | | | | | 1 | | | | | -1 | | | | | -1 | | | |
|  |  | MPS HAQ-Stairs* | | 0 | | 0 | | | 0 | | | 0 | | | 0 | | | 0 | | Sleep/Rest Fatigue | | 0 | | | | 0 | | | | | | 0 | | | | | | 0 | | | | | | -17 | | | | | | -8 | | MPS HAQ-Dressing* | | | | 1 | | | | 1 | | | | | 0 | | | | | 1 | | | | | 1 | | | | | 1 | | | |
|  |  | MPS HAQ-Movement* | | 2 | | 2 | | | 0 | | | 1 | | | 0 | | | 0 | | Cognitive Fatigue | | -9 | | | | -38 | | | | | | -5 | | | | | | 17 | | | | | | 30 | | | | | | 25 | | MPS HAQ-Eating & Drinking* | | | | 2 | | | | 2 | | | | | 0 | | | | | 2 | | | | | 3 | | | | | 1 | | | |
|  |  | CHAQ-Pain* | | 20 | | 20 | | | 49 | | | -4 | | | 1 | | | 1 | |  | |  | | | |  | | | | | |  | | | | | |  | | | | | |  | | | | | |  | | MPS HAQ-Hygiene* | | | | 0 | | | | -1 | | | | | -1 | | | | | 0 | | | | | 0 | | | | | 0 | | | |
|  |  |  | |  | |  | | |  | | |  | | |  | | |  | |  | |  | | | |  | | | | | |  | | | | | |  | | | | | |  | | | | | |  | | MPS HAQ-Caregiver Assist* | | | | 3 | | | | 2 | | | | | 0 | | | | | -1 | | | | | -1 | | | | | 0 | | | |
|  | |  | |  | |  | | |  | | |  | | |  | | |  | |  | |  | | | |  | | | | | |  | | | | | |  | | | | | |  | | | | | |  | |  | | | |  | | | |  | | | | |  | | | | |  | | | | |  | | | | |  | | | |
| **D12**  **Male  25.3-yrs** | | 6MWT (m) | | -19 | | -57 | | | -51 | | | 65 | | | 65 | | | 65 | | Total Fatigue | | 6 | | | | 8 | | | | | | 4 | | | | | | -3 | | | | | | -7 | | | | | | -8 | | BOT2-Fine | | | | 6 | | | | 0 | | | | | 3 | | | | | -1 | | | | | 1 | | | | | -3 | | | |
|  |  | 2MWT (m) | | -7 | | -22 | | | -19 | | | 21 | | | 24 | | | 20 | | General Fatigue | | -20 | | | | -12 | | | | | | -33 | | | | | | -4 | | | | | | -4 | | | | | | -4 | | BOT2-Dexterity | | | | 2 | | | | 5 | | | | | 1 | | | | | 2 | | | | | 1 | | | | | 2 | | | |
|  |  | CPE-Walking* | | 1 | | 3 | | | 2 | | | -1 | | | -2 | | | -1 | | Sleep/Rest Fatigue | | 25 | | | | -4 | | | | | | 38 | | | | | | -13 | | | | | | -17 | | | | | | -17 | | MPS HAQ-Dressing* | | | | 0 | | | | 0 | | | | | 0 | | | | | 0 | | | | | 0 | | | | | 0 | | | |
|  |  | MPS HAQ-Walking* | | -2 | | 0 | | | -4 | | | 2 | | | 2 | | | 2 | | Cognitive Fatigue | | 12 | | | | 41 | | | | | | 8 | | | | | | 8 | | | | | | 0 | | | | | | -4 | | MPS HAQ-Eating & Drinking* | | | | 0 | | | | 0 | | | | | 0 | | | | | 0 | | | | | 0 | | | | | 0 | | | |
|  |  | 3MSC (Steps) | | 17 | | -31 | | | -43 | | | 60 | | | 21 | | | 21 | |  | |  | | | |  | | | | | |  | | | | | |  | | | | | |  | | | | | |  | | MPS HAQ-Hygiene* | | | | 0 | | | | 0 | | | | | 0 | | | | | 0 | | | | | 0 | | | | | 0 | | | |
|  |  | MPS HAQ-Stairs* | | 0 | | 0 | | | -2 | | | 1 | | | 2 | | | 0 | |  | |  | | | |  | | | | | |  | | | | | |  | | | | | |  | | | | | |  | | MPS HAQ-Caregiver Assist* | | | | 0 | | | | 0 | | | | | 0 | | | | | 0 | | | | | 0 | | | | | 0 | | | |
|  |  | BOT2-Running | | -3 | | -6 | | | -13 | | | 6 | | | 7 | | | 8 | |  | |  | | | |  | | | |  | | | | | |  | | | | |  | | | | | |  | | | | |  | | | |  | | | | |  | | | | |  | | | | |  | | | | |  | | | | |  | | |
|  |  | CPE-Running* | | 1 | | 1 | | | 2 | | | -2 | | | -3 | | | -1 | |  | | \|  \|  \|  \|  \|  \|  \|  \| \| --- \| --- \| --- \| --- \| --- \| --- \| --- \|   Worsening Improving | | | |  | | | |  | | | | | |  | | | | |  | | | | | |  | | | | |  | | | |  | | | | |  | | | | |  | | | | |  | | | | |  | | | | |  | | |
|  |  | BOT2-Balance | | -1 | | 7 | | | -1 | | | -2 | | | 3 | | | 3 | |  | |  | | | |  | | | |  | | | | | |  | | | | | Cells right of the green line- vestronidase alfa; Cells left of the green line – placebo | | | | | |  | | | | |  | | | |  | | | | |  | | | | |  | | | | |  | | | | |  | | | | |  | | |
|  |  | CPE-Gross* | | 2 | | 2 | | | 3 | | | -1 | | | -3 | | | -1 | | Cells right of the green line- vestronidase alfa; Cells left of the green line – placebo   \|  \|  \|  \|  \|  \|  \|  \| \| --- \| --- \| --- \| --- \| --- \| --- \| --- \|   Worsening Improving | |  | | | |  | | | |  | | | | | |  | | | | |  | | | | | |  | | | | |  | | | |  | | | | |  | | | | |  | | | | |  | | | | |  | | | | |  | | |
|  |  | MPS HAQ-Movement* | | 0 | | 0 | | | -1 | | | 1 | | | 1 | | | 1 | |  | |  | | | |  | | | |  | | | | | |  | | | | |  | | | | | |  | | | | |  | | | |  | | | | |  | | | | |  | | | | |  | | | | |  | | | | |  | | |
|  |  | PROMIS-Pain* | | 0 | | 50 | | | 25 | | | 25 | | | 20 | | | 0 | |  | |  | | | |  | | | |  | | | | | |  | | | | |  | | | | | |  | | | | |  | | | |  | | | | |  | | | | |  | | | | |  | | | | |  | | | | |  | | |

**Footnote for Supplementary Figure 1.** *The negative changes (from randomization for placebo period and from baseline for UX003 period) are displayed since decreases in these scores indicate improvement. 6MWT, 6-Minute Walk test; 2MWT, 2-Minute Walk test; CPE, Clinical Problem Evaluation; MPS HAQ, MPS Health Assessment Questionnaire; 3MSC (Steps), 3-Minute Stair Climb test; BOT2, Bruininks-Oseretsky Test of Motor Proficiency; CHAQ, Childhood Health Assessment Questionnaire; PROMIS, PROMIS Health Assessment Questionnaire
